# The effect of perceived organizational support on organizational citizenship behavior and organizational commitment in public health center during COVID-19 pandemic

**DOI:** 10.3389/fpsyg.2022.938815

**Published:** 2022-08-01

**Authors:** Arif Firmansyah, I. Wayan Ruspendi Junaedi, Anang Kistyanto, Misbahuddin Azzuhri

**Affiliations:** ^1^Department of Management, Universitas Airlangga, Surabaya, Indonesia; ^2^GITM, National Chung Hsing University, Taichung, Taiwan; ^3^Department of Management, Universitas Dhyana Pura, Bali, Indonesia; ^4^Department of Management, Universitas Negeri Surabaya, Surabaya, Indonesia; ^5^College of Management, Yuan Ze University, Taoyuan, Taiwan; ^6^Department of Management, Universitas Brawijaya, Malang, Indonesia

**Keywords:** perceived organizational support, organizational citizenship behavior, organizational commitment, public health center, COVID-19 pandemic

## Introduction

The government reports that the number of COVID-19 cases in Indonesia has increased by 174 cases. Thus, since the beginning of the pandemic, the number of COVID-19 sufferers in Indonesia has currently reached 6,052,764 cases. This data contains details of 5,893,340 people whom have been declared cured (97.37%) and 156,534 people who died (2.59%), while the rest are still undergoing treatment. According to Dinc et al. (2022) the transmission of the COVID-19 virus is quite significant because it spreads globally, including in Indonesia. The first case occurred in February and as of September 30, 2020, the number of COVID-19 cases has reached 287,000 patients. In 11 days after the first case was announced, the number of positive cases reached 69 people, 4 of whom died and 5 recovered. Vu et al. (2022) stated the rapid development of this case has forced the government to issue various policies that are considered to be able to suppress its spread. The central government has issued a disaster emergency status starting from 29 February 2020 to 29 May 2020 regarding this virus, with a total time of 91 days (2). Some of the policies implemented are limiting activities outside the home, dropping out of school activities, and discouraging worship activities. However, in reality, there is disharmony between the policies made by the Central Government and the policies of the Regional Governments at the beginning of the pandemic. This difference began when several regions implemented a lockdown policy while the central government set a Large-Scale Social Restriction (PSBB) policy. Although there are differences, these various policies have made people's mobility patterns at home and in various public places different (3). This affects the rate of spread of COVID-19.

Oubibi et al. (2022) declare health services are currently the main focus. Improving the quality of service quality is starting to be improved in various aspects. Population health is the goal of every country, improvements to improve the quality of health are carried out in various ways, such as increasing awareness, willingness, and the ability to live a healthy life for everyone in order to realize the highest degree of public health. Through good health quality will affect the quality of human resources in the future. This shows that the health of the population still needs more serious attention from all parties, because its impact can affect the quality of human resources in the future. Efforts to support and achieve Government programs to make it happen through health service institutions commonly referred to as community health centers. To achieve this goal various work programs have been implemented.

The Ministry of Health seeks to improve public access to health services quality, especially those that can be realized through a service institution that has the aim of improving the health status of the community by ensuring the availability of health that is complete, equitable, high quality, and just. With increasing public awareness, the demand for quality services is also increasing and this means that Public health workers are also required to always improve their abilities and skills and attitudes work in order to provide the best service to the community. Achieving company goals is the desire of every employee, it is also desired by health service institutions such as health centers. To achieve it Employee volunteerism is needed in helping other employees in carrying out their duties, doing employee work for others when their work piles up as well as other jobs related to the demands of the internal work program to improve the quality of health or what is commonly called organizational citizenship behavior (OCB). To win the tight business competition, the organization not only requires employees to work optimally according to what is assigned or according to their job descriptions, but also expects employees who are willing to work outside their job descriptions. Employee behavior outside the job description is also known as organizational citizenship behavior (OCB).

## Organizational citizenship behavior

Organization Citizenship Behavior (OCB) is a voluntary behavior to help others exceed the demands of the role in the workplace or being organized and is not rewarded by the achievement of task performance. In order for OCB to be well-formed, there are three motives that underlie the purpose of these motives, namely the achievement motive, affiliation, and power. The motivation for employee achievement is one's effort to help other employees do their work in order to produce achievement for the success of the task of providing antenatal care services. Employee affiliation motive is a person's social behavior to improve relationships with others in providing antenatal care services. The motivation for employee power is a condition that encourages employees to seek status according to their main duties and functions so that the work is more focused on the maximum with better results. Good job status can motivate and control the work of others in providing antenatal care services. Based on the results of the study, the motive for power is the most dominant motive in forming OCB in Public health centers. Altruism is the attitude or behavior of helping others voluntarily and without coercion in carrying out tasks related to the organization for both the employees themselves and the organization in providing antenatal care services. Sportsmanship is the behavior of maintaining good relations with colleagues in order to avoid interpersonal problems in providing antenatal care services. Civic virtue is behavior that indicates responsibility in organizational life in providing antenatal care services. Courtesy is the behavior of maintaining good relations between employees, superiors, and service recipients so that services are good and avoid organizational interpersonal problems in providing antenatal care services.

According to Desky et al. (2020), Kotamena et al. (2020), and Francisco and Saoloan (2021) Organizational Citizenship Behavior (OCB) is individual behavior that is not directly recognized by the formal reward system which will have an impact on more effective organizational functions. The same opinion was added by Praditya (2020) and Purwanto (2022) defines OCB as behavior that goes beyond Sa'adah and Rijanti (2022) stated that organizational citizenship behavior is a preferred behavior that is not part of an employee's formal work obligations, but supports the effective functioning of the organization. Sumarsi and Rizal (2021) state citizenship behavior (OCB) is a beneficial behavior carried out by employees, independently of their provisions or obligations with the aim of achieving organizational goals. In addition, Supriadi et al. (2020) identified five OCB indicators, (1) Altruism, (2) Conscientiousness, (3) Sportsmanship, (4) Courtesy, and (5) CivicVirtue.

## Perceived organizational support

Perceived Organizational Support (POS) is a member's perception of the extent to which the organization values their contribution and cares about their well-being. While Supriadi et al. (2020) stated that perceived organizational support is defined as the perception of members about the extent to which the organization provides support to employees and the extent to which the organization is ready to provide assistance when needed. According to Sumarsi and Rizal (2021), organizational support is a situation where employees feel appreciated by their organization for their contributions, and that the company is looking out for their welfare. Nadeak et al. (2021) and Purwanto (2022) identified three indicators of perceived organizational support, (1) Fairness, (2) Supervisor support (Supervisor Support), and (3) Organizational Rewards and Working Conditions (Organizational Support). Rewards and Job Conditions).

## Organizational commitment

According to Prayuda (2019) and Sa'adah and Rijanti (2022), organizational commitment is defined as an attitude that shows employee loyalty and is a person's continuous process of expressing their concern for organizational success. Supriadi et al. (2020) and Suprapti and Rizal (2022) also have the same opinion, that commitment is a strong desire to remain as a member of a particular organization, a desire to strive according to the wishes of the organization, and a belief in the acceptance of organizational values and goals. There are three indicators used to measure the organizational commitment variable using a measurement scale developed by Kotamena et al. (2020) and Francisco and Saoloan (2021) (1) Affective Commitment (2) Continuing Commitment and (3) Normative Commitment.

## Result and discussion

### The effect of perceived organizational support on organizational commitment

Perceived organizational support has a positive effect on organizational commitment is accepted. This means that perceived organizational support on the influence of perceived organizational support on organizational citizenship behavior with nurse commitment still needs to be improved. Nurses who feel valued and get justice, feel comfortable with working conditions and atmosphere, and get support from their co-workers, will have an impact on nurses' comfort at work, so that it will increase nurse commitment. When employees feel the organization supports and cares about their welfare, employees will survive and remain loyal to the hospital where they work. Sumarsi (2019), Supriadi et al. (2020), Sumarsi and Rizal (2021), and Suprapti and Rizal (2022) explain that if employees feel the support from the organization and that support is in accordance with their norms, desires, and expectations, the commitment they have is likely great and can increase. According to Ahmadi et al. (2020) and Asbari et al. (2020) in his research stated the perception of organizational support consisting of: support from superiors, awards, and working conditions that have been given and provided by the organization will be able to make employees comfortable and at home to stay and work in the organization. Capone et al. (2022) with the comfort and convenience of employees living in the organization, then the commitment to stay in the organization will be strong along with the comfort and harmony of existing values that are felt to be in accordance with what is expected by each employee. The results of this study are in line with research conducted by Prayuda (2019), Praditya (2020), Supriadi et al. (2020), and Sa'adah and Rijanti (2022) which states that perceived organizational support has a positive and significant relationship to organizational commitment. This study states that when what is expected is in accordance with their aspirations, employees will have a high commitment to where the employee works. According to Supriadi et al. (2020) also stated that organizational support has a positive and significant effect on organizational commitment. The better or higher the organizational support provided by the company, the more it will increase the employee's organizational commitment to the company.

### The effect of organizational commitment on organizational citizenship behavior

Organizational commitment has a positive effect on organizational citizenship behavior is accepted. So it can be concluded that high organizational commitment by nurses will tend to increase nurses' organizational citizenship behavior. Nurses who feel that they are part of the organization, wish to spend the rest of their careers in the organization. Feeling bound both emotionally and financially, and feeling obligated to the organization will encourage nurses to work beyond their job descriptions in an effort to advance the hospital where they work. When a sense of belonging and loyalty as a reflection of organizational commitment is shown by employees to an organization, then the organizational citizenship behavior of employees has an increasing trend which can be seen from the positive behaviors shown by employees toward the organization. In line with these studies, Ahmadi et al. (2020), Asbari et al. (2020), and Desky et al. (2020) also state that organizational commitment has a positive and significant effect on organizational citizenship behavior variables. This means that the better or higher the employee's organizational commitment, the higher the employee's organizational citizenship behavior. The results of this study are in line with research conducted by Sumarsi and Rizal (2021) and Sa'adah and Rijanti (2022) which stated that organizational commitment has a positive and significant relationship to organizational citizenship behavior. Another research by Supriadi et al. (2020) and Suprapti and Rizal (2022) also explain that there is a significant relationship between organizational commitment and organizational citizenship behavior.

### The effect of perceived organizational support on organizational citizenship behavior

Perceived organizational support has a positive effect on organizational citizenship behavior is accepted. So it can be concluded that high perceived organizational support by nurses will tend to increase nurses' organizational citizenship behavior. Wuttaphan (2022) alleged that perceived organizational support provides assurance to employees that they receive benefits from leaders by showing OCB. Increased perceived organizational support can make employees feel obliged to contribute and care about the welfare of the organization as well as assist the organization in achieving its goals. According to Kotamena et al. (2020) and Nadeak et al. (2021) the greater the perceived organizational support felt by employees, the more employees will voluntarily make extra efforts for the benefit of their organization. The results of this study are in line with research conducted by Sumarsi (2019) and Sumarsi and Rizal (2021) which states that the perception of organizational support has a positive and significant effect on organizational citizenship behavior. in line With this, Supriadi et al. (2020) also explain that the better employees feel that they get organizational support in the environment where they work, the higher the organizational citizenship behavior in question.

### The effect of perceived organizational support on organizational citizenship behavior through organizational commitment

Perceived organizational support variable on organizational citizenship behavior has a significant effect through organizational commitment. This shows that the organizational commitment variable is able to increase perceived organizational support for nurses' organizational citizenship behavior and has a positive mediating effect. So it can be concluded that perceived organizational support by nurses can have a direct influence on nurses' organizational citizenship behavior, but the effect will be better if perceived organizational support is also followed by an indirect effect of organizational commitment. The results of this study are in line with research conducted by Sumarsi (2019), Supriadi et al. (2020), Sumarsi and Rizal (2021), and Suprapti and Rizal (2022) which states that the organizational commitment variable has a role as a mediating/intervening variable in the relationship between perceptions of organizational support for organizational citizenship behavior. Ahmadi et al. (2020) stated that affective commitment mediates the relationship between perceived organizational support and organizational citizenship behavior indirectly.

### Recommendation

Based on the results of the analysis, recommendations were obtained for the development of OCB in order to improve organizational performance in providing antenatal care services in Banyuwangi Regency. These recommendations relate to improving the quality of interaction between superiors and subordinates as well as the culture and climate of the organization. Recommendations to improve the quality of interaction with subordinates are: 1) Superiors and subordinates evaluate each other on the implementation of work in meetings (subordinates provide proposals and superiors provide evaluations); 2) There must be good coordination between superiors and subordinates in the form of reports from subordinates to superiors; 3) Supervisors and subordinates regularly attend mini-workshops which are held every month to analyze performance results and problem-solving solutions; 4) Superiors give subordinates the freedom to innovate to improve performance; 5) Superiors always provide a clear picture to subordinates so that all programs can be achieved in accordance with the expected targets. This is done by always providing input or advice if something is less than the target throughout a meeting. Recommendations to improve organizational culture and climate are: 1) Leaders always remind the mutually agreed commitments to improve services to the community; 2) Leaders always provide opportunities for employees to be creative and innovate in carrying out their duties; 3) Employees always come when invited to regular meetings; 4) Leaders always remind to work well in teams; 5) Leaders always remind commitment in providing services; 6) Leaders and employees establish a mutual agreement in each service improvement plan.

## Conclusion

Based on the research objectives, problem formulation, and research results with the discussion that has been described, conclusions can be drawn from this study as follows: 1) There is a direct and significant, and positive effect between perceived organizational support and organizational commitment to nurses. This means that nurses feel that organizational support given is able to influence how they want to survive and be loyal in the hospital where nurses work. 2) There is a direct and significant and positive effect between organizational commitment and organizational citizenship behavior on nurses. This means that:

Having high organizational commitment will be able to influence how they want to work outside their job description (extra-role behavior). This is because if a nurse has a high commitment to the hospital where she works, then the nurse tends to display a positive attitude toward work and organization. This positive attitude encourages employees to have a willingness to be involved in work even outside of their job descriptions, this will be in contrast to nurses with low organizational commitment, so nurses will be reluctant to work outside their job descriptions. 3) There is a direct and significant and positive effect between perceived organizational support and organizational citizenship behavior on nurses. This means that nurses feel that the organizational support provided is able to influence how they want to work outside their job descriptions in an effort to advance the hospital. There is an indirect and significant and positive effect between perceived organizational support and organizational citizenship behavior through an organizational commitment to nurses. This means that when perceived organizational support is high, organizational commitment will also be high, with nurses having a high organizational commitment, then there is a desire for these nurses to increase positive behaviors related to organizational citizenship behavior.

## Author contributions

All authors listed have made a substantial, direct, and intellectual contribution to the work and approved it for publication.

## Conflict of interest

The authors declare that the research was conducted in the absence of any commercial or financial relationships that could be construed as a potential conflict of interest.

## Publisher's note

All claims expressed in this article are solely those of the authors and do not necessarily represent those of their affiliated organizations, or those of the publisher, the editors and the reviewers. Any product that may be evaluated in this article, or claim that may be made by its manufacturer, is not guaranteed or endorsed by the publisher.
